# Aprotic Solvent Accumulation Amplifies Ion Current Rectification in Conical Nanopores

**DOI:** 10.1021/acs.jpcb.2c03172

**Published:** 2022-07-22

**Authors:** Emer B. Farrell, Dominik Duleba, Robert P. Johnson

**Affiliations:** School of Chemistry, University College Dublin, Belfield, Dublin 4 D04 V1W8, Ireland

## Abstract

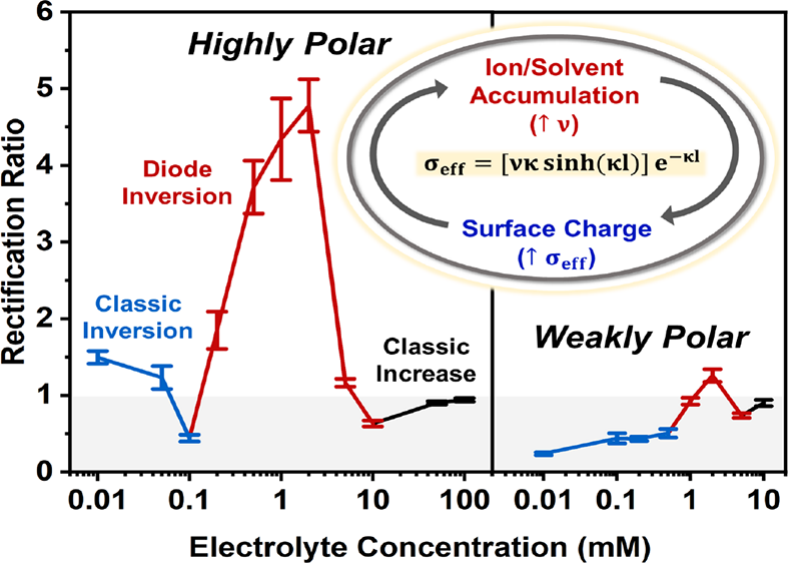

Ion current rectification is highly reported in aqueous electrochemical systems and sensors but lacks exploration in organic systems due to the additional complexity introduced by non-aqueous solvents. Herein, a detailed study on ion current rectification with highly polar and mildly polar aprotic organic solvents as a function of tetraethylammonium tetrafluoroborate supporting electrolyte concentration is presented. To explain our experimental results, we introduce a previously unreported phenomenon: the formation of a double-junction diode within the nanopore that arises due to a complex interplay between ion and solvent enrichment effects. Finite element simulations are used to explore this phenomenon and the subsequent effect on the rectifying behavior of conical quartz nanopores.

## Introduction

Ion current rectification (ICR) is a phenomenon in which a current–voltage trace exhibits non-ohmic behavior and the current measured at a potential is unequal to that measured at the opposite potential.^1,2^ Positive ICR occurs when the current measured at positive potentials is greater than that at negative potentials, while negative ICR implies the opposite. This phenomenon occurs only when nanopores exhibit an asymmetry that can facilitate the necessary electrical double layer (EDL) overlap and perm selectivity at the nanopore tip.^3^ ICR is usually a result of an asymmetric conical/pyramidal pore geometry, but it also occurs in symmetric cylindrical pores that possess an asymmetric surface charge.^4,5^ Although asymmetry is a prerequisite for ICR to be observed, maximum rectification does not always occur at the largest EDL overlap as many complex ion-transport properties occur inside and outside of the nanopore tip. Several models for ICR, focusing predominantly on aqueous systems, have been proposed. Woermann described ICR in terms of transference numbers at the tip where ion enrichment/depletion due to EDL overlap gives rise to high and low conductivity states.^6^ Siwy et al.^7,8^ considered the formation of an electrostatic ion trap at a given applied potential along the length of the nanopore where EDL overlap occurs, again resulting in high and low conductivity states. Cervera et al.^9,10^ solved the Poisson–Nernst–Planck equations to demonstrate ICR theoretically. Numerous fundamental studies into the rectifying behavior of nanopores in aqueous electrolytes have been carried out, notably on the effect of variables including the electrolyte concentration and scan rate on the degree and direction of rectification.^11−13^ Changes in ICR upon interaction with an analyte can be employed practically in sensing applications and are described by Duleba et al.^14^ in a recent review paper. An interesting example, reported by Heaton and Platt, is a multiuse nanopore platform whose rectifying behavior is changed by placing a sheet of metal-immobilized paper on top of the nanopore.^15^ While aqueous sensors are suitable for environmental^16−19^ and biological applications,^20−22^ they have limited use in industrial settings that feature aprotic solvent production processes, such as in pharmaceutical plants.^23^ A deeper understanding of the rectifying behavior of nanopores in non-aqueous electrolytes is essential in the development of ICR sensors compatible with organic solvents and will facilitate a wider range of applications than that for which such sensors are currently developed.^14^

The ion current rectification of conical nanopores featuring an organic electrolyte is both less explored and more complex than for an aqueous electrolyte. Fundamental studies by Plett et al.^24^ and Yin et al.^25^ indicated a significant deviation in the rectifying behavior of nanopores in aprotic organic solvents compared to water, where the degree of ICR is dependent on the polarity of the solvent used. Both authors attributed this to the generation of an effective positive surface charge by the adsorption of solvent molecules to the nanopore wall.^24,25^ Subsequently, in mildly polar aprotic organic solvents, nanopores exhibit a lesser degree of rectification than in highly polar aprotic organic solvents.

Our group has previously reported the inversion of rectification at low electrolyte concentrations in aqueous KCl systems both experimentally and theoretically, and we explained these phenomena through changes in the position of ion enrichment and depletion peaks with the widening EDL.^13^ Following on from this study and inspired by the work of Plett et al.^24^ that demonstrated solvent-induced positive surface charges in aprotic solvent, we sought to investigate if an inversion of rectification at low electrolyte concentrations was observable in non-aqueous systems using highly polar (acetonitrile) and mildly polar (dichloromethane) aprotic organic solvents with tetraethylammonium tetrafluoroborate (TEATFB) as the supporting electrolyte. However, unexpectedly, we found extreme changes to the rectification behavior of the aprotic solvent containing pore, even at moderately low ionic strengths, that could not adequately be explained using existing models for ICR. Herein, we propose that these unexpected results arise from a previously unreported phenomenon: amplification of the ICR effect through solvent accumulation within the pore and the formation of a double-junction diode. Our hypothesis is supported by finite element simulations with COMSOL Multiphysics using the Poisson–Nernst–Planck–Navier–Stokes equations, which qualitatively replicate the significant inversions in the ICR magnitude and directionality as a function of the electrolyte concentration.

## Materials and Methods

### Materials and Reagents

Quartz capillaries (0.7 mm i.d., 1 mm o.d., Sutter Instruments) were used in the fabrication of the quartz nanopipettes. The electrolyte employed in organic ICR experiments was tetraethylammonium tetrafluoroborate (99%, Alfa Aesar) dissolved in acetonitrile (99.9%, Fisher Scientific) and dichloromethane (99%, Fisher Scientific). Nanopipette radii were measured using potassium chloride (99%, Acros Organics) dissolved in Milli-Q water with Ag/AgCl wires (prepared using Ag wires (99.9%, Merck)) as working and reference electrodes. Pt wires (99.9%, Merck) were used as electrodes in organic electrolyte systems. All current–voltage traces were measured using a Biologic SP-200 potentiostat fitted with an ultralow current option and high-speed scan. Measurements were performed with a filter band width of 50 kHz, and a moving average filter (window size 11 points) was applied after measurement using EC-Lab software to filter the noise numerically.

### Fabrication of Nanopipettes

Quartz capillaries were rinsed with deionized water and ethanol and dried overnight in an oven at 80 °C. Once dried, nanopipette fabrication was carried out using a Sutter P-2000 micropipette puller with five tunable parameters, namely, heat (*H*), filament (*F*), velocity (*V*), delay (*D*), and pull (*P*). The following program was employed to fabricate 50 nm nanopipettes: line 1: H700, F4, V20, D170, and P0 and line 2: H680, F4, V50, D170, and P200.

### Characterization of Nanopipettes

Nanopipette radii were determined by recording current–voltage traces using a 0.1 M KCl electrolyte in deionized water. Nanopipettes were back-filled with electrolyte, and a Ag/AgCl wire working electrode was inserted. The nanopipettes were placed in a bulk electrolyte bath containing a Ag/AgCl wire reference electrode such that the tip was submerged, and current–voltage traces were measured. The applied potential was swept from −0.6 to 0.6 V with respect to the reference electrode at a scan rate of 0.1 V s^–1^. A linear fit was applied to the resulting CV using EC-Lab software, and the slope was used to calculate the nanopipette radius based on eq 1.^26,27^
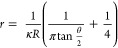
1where κ is the electrolyte conductivity, θ is the cone angle, and *R* is the nanopipette resistance. By inserting resistance, the inverse of conductivity (obtained from the slope of the CV), the nanopipette radius (*r*) can be determined, assuming a constant cone angle between nanopipettes and excluding the effect of the nanopipette wall surface charge.

### ICR Measurements

Nanopipettes were backfilled with tetraethylammonium tetrafluoroborate (TEATFB) of varying concentrations in acetonitrile (MeCN) and dichloromethane (DCM). A Pt wire working electrode was inserted into the nanopipettes, which were placed in a bulk electrolyte bath of the same concentration containing a Pt wire reference electrode. Current–voltage traces were measured using a Biologic SP-200 potentiostat with an ultralow current probe. The applied potential was swept from −1 to 1 V with respect to the reference electrode at a scan rate of 0.1 V s^–1^. Experimental error bars were calculated from at least five data points as the standard error. Sample CVs are shown in Figure S3.

### Finite Element Simulations

COMSOL Multiphysics 6.0 was used to solve Poisson–Nernst–Planck and Navier–Stokes equations, using the following physics modules: transport of diluted species (tds), electrostatics (es), and creeping flow (spf). The basic nanopipette geometry was modeled as 2D axisymmetric with a pipette height of 5 μm, a pipette radius of 50 nm, and a half-cone angle of 10°, shown in Figure S1. The bulk electrolyte was modeled as a square of width of 2.5 μm. A region for finer meshing of the electrical double layer (EDL) with a width of 5 nm was input as well as a small rectangular region at the nanopipette tip with a height of 5 nm. The nanopipette wall was assumed to have a width of 2 nm. Mesh refinement was performed until there was no change in RR with a decreasing element size. The meshing is shown in Figure S1. Boundary conditions were applied at the nanopipette wall, interior bulk solution, and exterior bulk solution as shown in Table S1 and Figure S1. The dielectric constants for MeCN and DCM were taken to be 37.5 and 8.93, respectively. The diffusion coefficients were based on reported values where NEt_4_^+^ was 0.96 × 10^–9^ m^2^ s^–1^ and BF_4_^–^ was 0.82 × 10^–9^ m^2^ s^–1^.^28^

The Nernst–Planck equation was used to simulate the flux of ions arising from diffusion, migration, and convection:
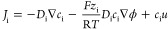
2where *J*_i_ is the flux of an ion, *D*_i_ is the diffusion coefficient of an ion, *c*_i_ is the ion concentration, *z*_i_ is the ion charge, R is the ideal gas constant, *T* is the temperature, ϕ is the electric potential, and *u* is the fluid velocity.

The Poisson equation was used to solve the distribution of the electric field:
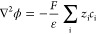
3where ϕ is the electric potential, ε is the dielectric permittivity, *z*_i_ is the ion charge, and *c*_i_ is the ion concentration.

The Navier–Stokes equation was used to solve the fluid velocity and pressure distribution:
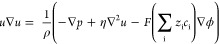
4where *u* is the fluid velocity, ρ is the solvent density, η is the solvent viscosity, *p* is the applied pressure, *z*_i_ is the ion charge, *c*_i_ is the ion concentration, and ϕ is the electric potential.

## Results and Discussion

### ICR in a Weakly Polar Organic Solvent

**Figure 1:**
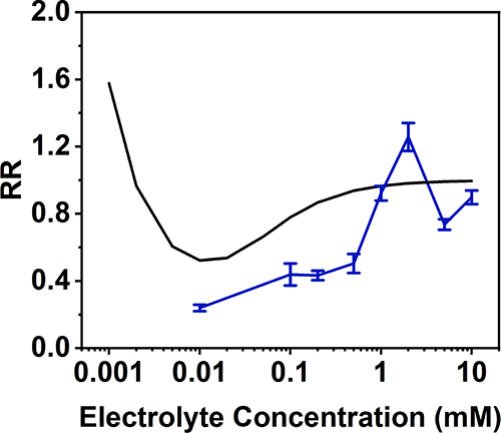
Experimental data (blue) and theoretical data (black) showing the change in the rectification ratio (RR) with a decreasing electrolyte concentration (mM) in dichloromethane (DCM) in 50 nm glass nanopipettes using tetraethylammonium tetrafluoroborate (TEATFB) as the supporting electrolyte. All theoretical calculations assumed a uniform surface charge distribution of 0.1 mC m^–2^ on the nanopore surface.

Figure 1 shows the experimental rectification ratio (RR), obtained from current–voltage curves using eq 5 at various concentrations of TEATFB in dichloromethane (DCM).
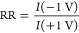
5The direction of rectification is generally positive in DCM, and it initially increases with a decreasing electrolyte concentration due to the widening of the EDL and the corresponding increasing perm selectivity at the tip. A small peak appears at around 1 mM where rectification inverts beyond unity before returning to its initial positive state. After this inversion, rectification continues to increase until reaching a maximum. The positive rectification observed has been attributed previously to the formation of a weak positive charge at the nanopore surface that arises from the orientation of the solvent layer at the nanopore surface with a positive dipole moment pointing outward into the solution.^24^ In Figure 1, the theoretical RR, calculated by assuming a nanopore surface charge of 0.1 mC m^–2^, is compared to the experimental RR values, showing a qualitative agreement except at concentrations around 1 mM. The theoretical results show a rectification inversion at <0.01 mM, similar to the rectification inversion observed in aqueous systems at low electrolyte concentrations and herein referred to as classical inversion.^13^ This inversion could not be collected experimentally as the conductivity of the solution was too low to give a measurable current.

### ICR in a Highly Polar Organic Solvent

**Figure 2:**
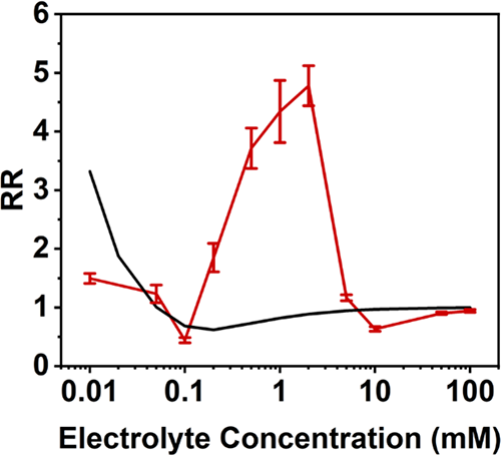
Experimental data (red) and theoretical data (black) showing the change in the rectification ratio (RR) with a decreasing electrolyte concentration (mM) in acetonitrile (MeCN) in 50 nm glass nanopipettes using tetraethylammonium tetrafluoroborate (TEATFB) as the supporting electrolyte. All theoretical calculations assumed a uniform nanopore surface charge distribution of 1 mC m^–2^.

Figure 2 shows the experimental change in the RR with a decreasing concentration of TEATFB in acetonitrile (MeCN). The experimental results are similar to those obtained for DCM, although the rectification inversion circa 1 mM is fivefold larger in magnitude. The classical rectification inversion occurs at a higher electrolyte concentration than in DCM, indicating that the surface charge in the MeCN system is larger. This agrees with previous hypotheses by Plett et al.^24^ and Yin et al.^25^ relating solvent polarity to the degree of rectification. For the theoretical calculations, shown in Figure 2, the nanopore wall assumed a larger surface charge than in the DCM system, shown in Figure 1. This is in accordance with hypotheses by Plett et al.^24^ and Yin et al.^25^ stating that solvents of higher polarity impart a larger effective surface charge on the nanopore wall. Hence, in the theoretical calculations, shown in Figure 2, the nanopore wall assumed a uniform surface charge distribution of 1 mC m^–2^, giving close agreement at all concentrations outside of the 0.1 to 5 mM window where an extreme and unexpected rectification inversion above unity is experimentally observed.

**Figure 3:**
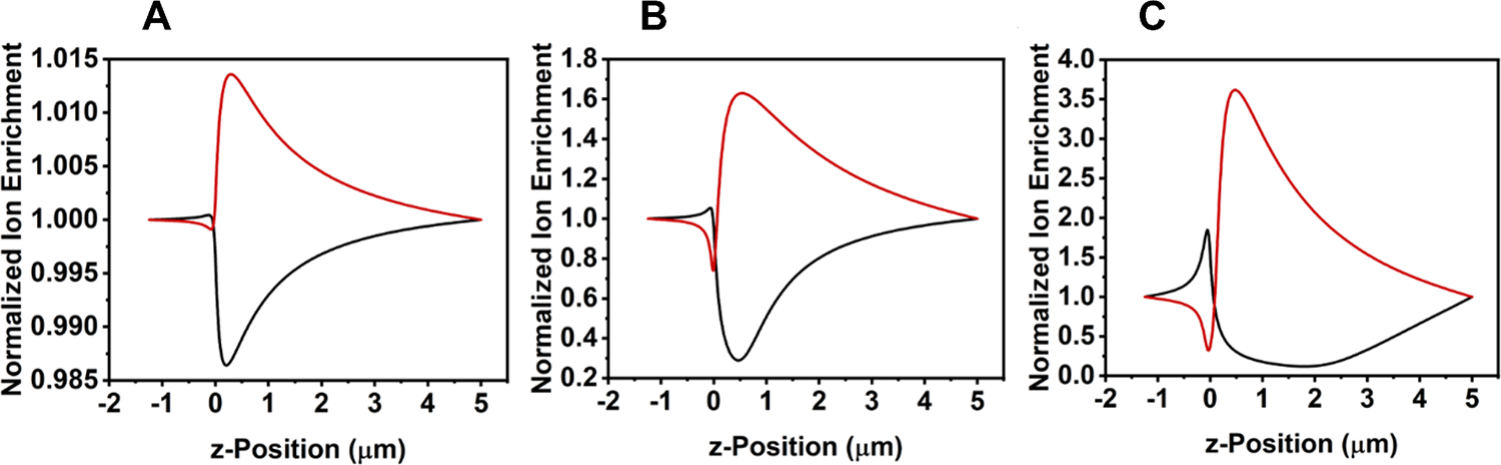
Simulated normalized cation enrichment ([TEA^+^]/[Bulk]) along the nanopipette central axis (*z*) at positive potential (red) and depletion at negative potential (black) at TEATFB concentrations of (A) 10 mM (pre-rectification maximum), (B) 0.1 mM (rectification maximum), and (C) 0.01 mM (post-rectification inversion) in MeCN using a 50 nm glass nanopipette, assuming a uniform surface charge of 1 mC m^–2^ on the nanopipette wall.

The rectification inversion that occurs at <0.1 mM in MeCN can be explained by plotting the normalized cation enrichment/depletion (taken as the concentration of TEA^+^ divided by the bulk electrolyte concentration) along the nanopipette central axis *z*. At large electrolyte concentrations (Figure 3A), both enrichment and depletion occur further inside the nanopipette, giving rise to classical rectification behavior (excluding 1 mM). At the rectification maximum (0.1 mM), ion enrichment and depletion peaks widen and shift deeper within the nanopore while counteracting enrichment/depletion peaks appear at the pore mouth, resulting in reaching a maximum in the positive rectification (Figure 3B). Finally, at the rectification inversion beyond unity (0.01 mM), the enrichment/depletion peaks at the nanopore tip become dominant over the enrichment/depletion peaks further within the pore, leading to inverse rectification (Figure 3C). This is in agreement with previous studies in aqueous systems.^13^

**Figure 4:**
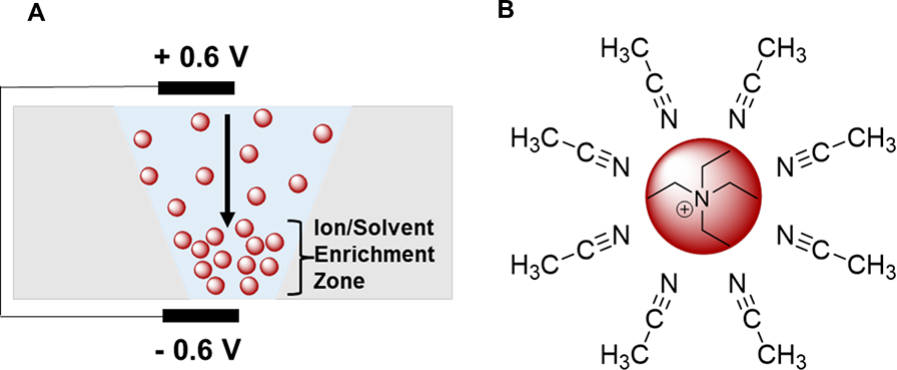
Schematic representations of (A) the accumulation of TEA^+^ cations in the nanopore tip, resulting in a region of solvent accumulation, at a positive applied potential. (B) TEA^+^ cation with an MeCN solvation sphere.

The deviation of the theoretical results from experimental data in MeCN and DCM indicates that modeling a uniform distribution of surface charge throughout the nanopipette is, unlike in aqueous nanopore systems, insufficient. Consequently, an effective positive surface charge due to dipole alignment cannot be considered to be solely responsible for the rectifying behavior of the nanopore. We postulated that the extreme rectification inversion observed between 5 and 0.1 mM could be the result of the formation of a double-junction diode in the nanopore. The double-junction diode forms as ions, carrying solvent molecules that directly impact the surface charge, accumulate/deplete in the nanopore at given applied potentials (Figure 4A). Plett et al.^24^ (eq 6) reported that the effective surface charge (σ_eff_) of a nanopore can be directly related to the dipole density (ν) in a layer of thickness (*l*) along the nanopore wall with an inverse Debye length (κ).
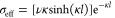
6

It follows that, in areas of ion enrichment, there is also an enrichment of solvent arising from the solvation shell, which, for a MeCN bulk solvent, contains 8 to 10 MeCN molecules (Figure 4B).^29−31^ The regions of the pore with solvent enrichment adopts a larger effective surface charge (σ_eff_) due to the larger dipole density (ν). As a result, in accumulation conditions at positive potential, the nanopore wall contains a region with a larger surface charge (accumulation diode), and in depletion conditions at negative potential, the nanopore wall contains a region with a lower surface charge (depletion diode). As the bulk electrolyte concentration changes, the position of these regions within the nanopore changes (Figure 3), facilitating sudden switches between positively and negatively rectifying states and enhanced rectification, similar to those previously reported in diodic nanopore systems.^32−35^ In aqueous systems, the solvent enrichment is not anticipated to be important because the surface charge of the nanopore is given by the protonation/deprotonation of surface groups and not by the solvent dipole alignment.

**Figure 5:**
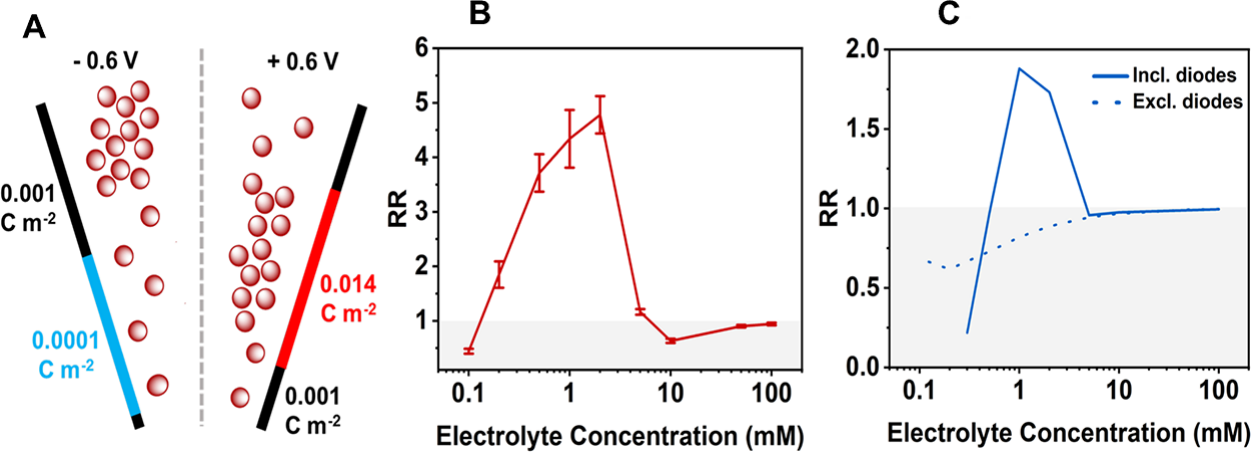
(A) Schematic of the accumulation and depletion double-junction diodes (at applied potentials of +1 and −1 V, respectively) in the theoretical model of rectification in MeCN at an electrolyte concentration of 1 mM. (B) Experimental and (C) theoretical data showing the RR as a function of the electrolyte concentration upon inclusion (solid) and exclusion (dashed) of the diode regions. All data is measured in 50 nm glass nanopipettes using solutions of TEATFB in MeCN as the bulk electrolyte.

The double-junction diode theory was implemented in the model by manually including a band of higher positive charge at positive potentials and a lower positive charge at negative potentials. This is schematically represented in Figure 5A at a single electrolyte concentration. The position of the bands was selected from the ion enrichment and depletion curves at each electrolyte concentration as obtained from the linear surface charge model (Figure 3). The double-junction diode boundaries at positive and negative potentials were selected as shown in Figure S2 and are summarized for each electrolyte concentration in Tables S2 and S3. Surface charge values were selected based on the height of the normalized enrichment/depletion peaks (Figure 3, Table S4, and Figure S2). A rectangle function with a smoothing factor of 300 nm was used to apply the non-uniform surface charge distribution onto the pore surface as sudden step changes in the surface charge prevent convergence of the simulation at high surface charge values. Figure 5B shows the experimental change in the RR as a function of the electrolyte concentration, compared to Figure 5C (Table S4) showing the theoretical change in RR with a decreasing electrolyte concentration upon the inclusion of accumulation and depletion diodes. The model achieved a significantly closer qualitative agreement to the experimental data than assuming a uniform surface charge distribution (Figure 5C) with the RR inverting beyond unity around 1 mM, suggesting that the double-junction diode theory is a viable explanation for the observed triple inversion in both the DCM and MeCN systems. It was also shown that the surface charge in the double-junction diode region has a significant effect on the direction and degree of rectification. Thus, the smaller inversion in the DCM system can be explained by a smaller surface charge on the nanopore wall due to its smaller dipole density (ν) in accordance with eq 6, as described by Plett et al.^24^

It is also important to consider a recent study by Polster et al.^36^ and an earlier study by Berne et al.^37^ where MeCN molecules have been shown to form lipid-like bilayers on silica surfaces, meaning that, in neat MeCN, the surface exhibits an effective negative charge. Polster et al.^36^ hypothesized that, at certain concentrations of lithium perchlorate (LiOCl_4_), the effective surface charge becomes positive due to the interaction of the supporting electrolyte ions with the MeCN bilayer. The outcome of the theoretical calculations assuming this hypothesis would remain the same however as an effective positive surface charge in the presence of a supporting electrolyte is assumed, the magnitude of which is affected by the concentration of ions in given regions at the silica surface.

While the theoretical model was able to qualitatively replicate the experimental data regardless of the surface charge applied in the accumulation and depletion regions (Figure S4), it was however unable to quantitatively account for extreme degrees of the double-junction diode inversion in MeCN (Figure 5B). The experimental data exhibits rectification ratios up to approximately threefold larger than observed in the simulations at the highest point (2 mM), and we attribute this to the inability of the model to converge at surface charges greater than 0.02 C m^–2^. We speculate that the double-junction diode operates in a positive feedback loop due to the mutual reliance of the surface charge on the degree of ion/solvent accumulation and vice versa (eq 6), meaning that the real surface charge in the accumulation diode must be significantly higher than is possible to incorporate into our simulations. Furthermore, the position of ion enrichment/depletion may also be dependent on the surface charge magnitude; therefore, the positions of the bands used in the model are a simplification and can introduce an error in the simulations. Additionally, when a dielectric material is exposed to an external electric field, a partially compensating electric field is produced within the material due to the polarization of atoms, resulting in an induced surface charge on the other side of the wall.^38,39^ Due to the intrinsic neutrality of the glass surface in the aprotic organic solvent, induced charge electro-osmosis (ICEO) may arise on the outer wall of the nanopipette, which will significantly impact the ICR observed in these systems.^40,41^ Developing a method to model this will allow for more accurate quantitative modeling of ICR in MeCN and DCM.

## Conclusions

This work presents for the first time the complex interplay between ion and solvent enrichment in conical nanopores in DCM and MeCN and its resulting effect on surface charge and ICR. At moderately low electrolyte concentrations, extreme inversions in the rectification direction are observed. We explain these observations through implementation of a previously undescribed double-junction diode theory where regions of increased (accumulation diode) and decreased (depletion diode) surface charges arise at positive and negative potentials due to the direct relationship between ion-associated solvent shell accumulation/depletion and the surface charge in an aprotic organic electrolyte. This theory is qualitatively supported by finite element simulations. This work is one of few publications exploring ICR in organic electrolytes and provides a vital in-depth understanding of the complex processes that arise in these systems. Understanding these systems is essential in expanding the scope of ICR-based nanopore sensors toward operation in organic electrolytes.

## Supporting Information Available

Additional information on the meshing and boundary conditions employed in the finite element simulations, selection of diode boundary regions and surface charges assumed in the accumulation and depletion diodes, sample CVs at each electrolyte concentration in MeCN, and additional theoretical data assuming different surface charges in the accumulation and depletion diodes (PDF)
