# Supplementary material for: Aprotic Solvent Accumulation Amplifies Ion Current Rectification in Conical Nanopores
Source: J Phys Chem B. 2022 Jul 22;126(30):5689–94. doi: 10.1021/acs.jpcb.2c03172 (PMC9358645; doi:10.1021/acs.jpcb.2c03172)
Supplement: Supplementary file 1 — jp2c03172_si_001.pdf [file jp2c03172_si_001.pdf]

Supporting Information

Aprotic Solvent Accumulation Amplifies Ion Current Rectification in Conical Nanopores

*Emer Farrell, Dominik Duleba, Robert P. Johnson**

School of Chemistry, University College Dublin, Belfield, Dublin 4, Ireland

*robert.johnson@ucd.ie

Table S1. The boundary conditions employed in finite element simulations corresponding to the geometry in Figure S1.

**Boundary**	**Nernst Planck (tds)**	**Navier Stokes (spf)**	**Poisson (es)**
**1. Interior Bulk**	Concentration cbulk	Inlet Pressure, 1 atm	Electric Potential Applied Potential
**2. Nanopipette Wall**	No Flux	No Slip	Surface Charge
**3. Exterior Bulk**	Concentration cbulk	Outlet Pressure, 1 atm	Ground

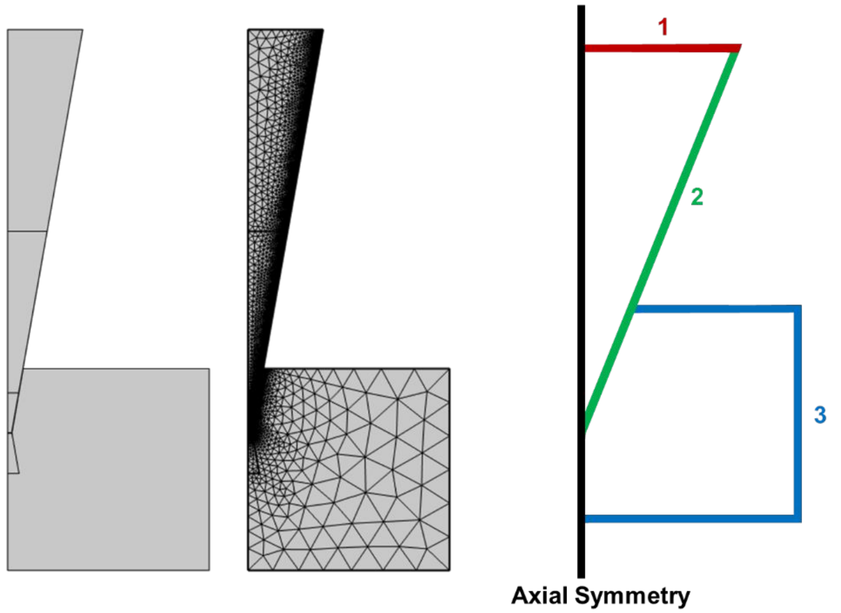

Figure S1. The 2D axisymmetric geometry, meshing and boundary conditions (corresponding to Table S1) employed in finite element simulations

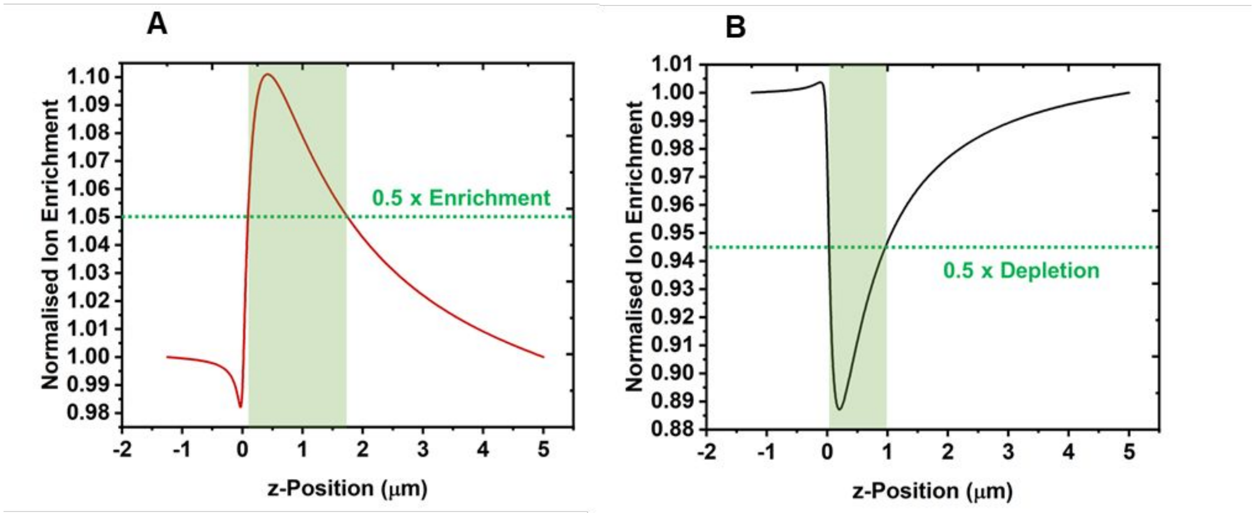

Figure S2. Selection of the boundaries for **A)** the accumulation and **B)** the depletion diode, based on the normalized ion enrichment and depletion curves, plotted along the central axis, z. The curves shown are at an electrolyte concentration of 0.1 mM, in MeCN, with a 50 nm nanopipette, assuming a surface charge of 1 mC m^{-2}.

Table S2. The boundaries of the accumulation diodes included in the COMSOL model

		**0.6 V Accumulation**	
**Conc. mM**	**Peak nm**	**Left boundary nm**	**Right Boundary**

			nm
100	200	20	1200
10	300	40	1400
1	400	90	1750
0.1	500	140	2100

*estimated using the procedure in Figure S2, at different electrolyte concentrations in MeCN and a 50 nm nanopipette, with an even surface charge of 1 mC m^{-2}.

Table S3. The boundaries of the depletion diodes included in the COMSOL model

		-0.6 V Depletion	
Conc. mM	Peak nm	Left boundary nm	Right Boundary nm
100	200	30	1050
10	200	25	1000
1	200	30	950
0.1	500	70	1350

*estimated using the procedure in Figure S2, at different electrolyte concentrations in MeCN and a 50 nm nanopipette, with an even surface charge of 1 mC m^{-2}.

Table S4. The simulated current at + and – 1 V, as a function of decreasing electrolyte concentration, and the corresponding surface charges used in the diodes

Conc.	SC 1 (C m^{-2})	SC 2 (C m^{-2})	SC 3 (C m^{-2})	I+ (A)	I- (A)	RR
100	0.001	0.002	0.0009	5.86E-09	-5.82E-09	0.99
10	0.001	0.002	0.0009	5.91E-10	-5.749E-10	0.97
5	0.001	0.002	0.0009	2.96E-10	-2.83E-10	0.96
2	0.001	0.014	0.0001	6.48E-11	-1.1229E-10	1.73
1	0.001	0.014	0.0001	2.89E-11	-5.42E-11	1.88
0.5	0.001	0.016	0.0001	2.63E-11	-2.539E-11	0.967
0.3	0.001	0.016	0.0001	5.60E-11	-1.22E-11	0.218

*calculated using the diode boundaries shown in Table S2 and S3. Data is recorded in a 50 nm nanopipette in MeCN. All simulations are performed with a rectangle function, with a smoothing factor of 300 nm. Surface Charge 2 (SC 2) refers to the surface charge in the accumulation diode at positive potential. Surface Charge (SC 3) refers to the surface charge in the depletion diode at negative potential. Surface Charge 1 (SC 1) refers to the surface charge on the rest of the nanopore walls. These are schematically represented in Figure 5A.

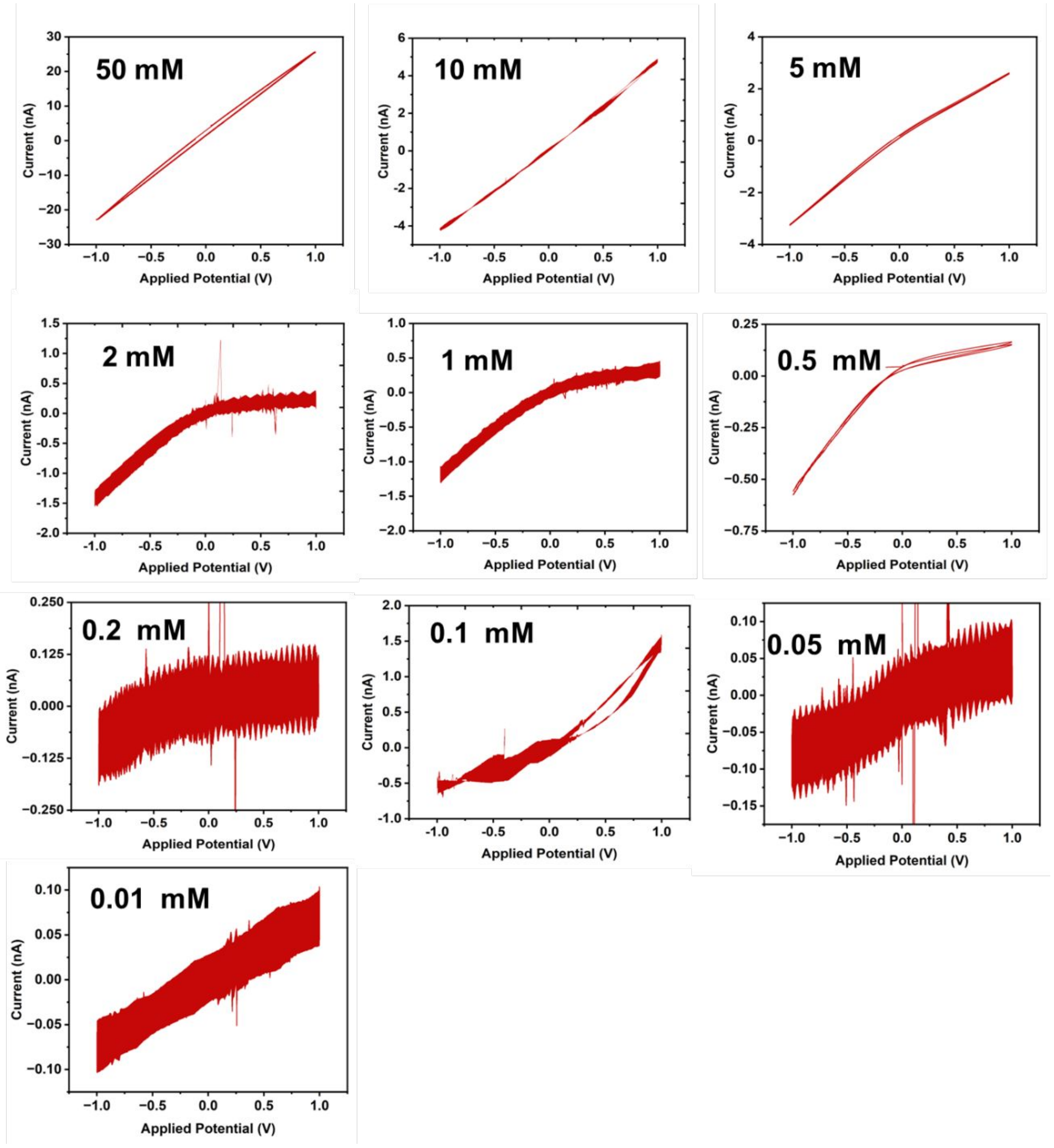

Figure S3. Cyclic Voltammograms at each electrolyte concentration, from which the RR values used in Figure 2 and Figure 5A are extracted. All measurements are recorded in 50 nm quartz nanopipettes, using TEATFB in MeCN as supporting electrolyte, and Pt wire electrodes. All CVs

are recorded at a scan rate of 0.1 V s^{-1}, with a filter bandwidth of 50 kHz, and a moving average filter (window size 11 points) applied post-measurement.

Table S5. The surface charges (C m^{-2}) used in the accumulation region (SC2) and depletion region (SC3) corresponding to Figure S4

	**SC2**	**SC2**	**SC2**	**SC2**	**SC2**	**SC2**
	**100**	**10**	**2**	**1**	**0.5**	**0.3**
**Green (5)**	0.002	0.003	0.004	0.005	0.006	0.008
**Blue (4)**	0.002	0.004	0.006	0.008	0.01	0.011
**Purple (3)**	0.002	0.003	0.006	0.01	0.012	0.015
**Red (2)**	0.002	0.003	0.012	0.016	0.018	0.019
**Black (1)**	0.002	0.002	0.014	0.014	0.016	0.016
	**SC3**	**SC3**	**SC3**	**SC3**	**SC3**	**SC3**
	**100**	**10**	**2**	**1**	**0.5**	**0.3**
**Green (5)**	0.0009	0.0008	0.0007	0.0006	0.0004	0.0002
**Blue (4)**	0.0009	0.0007	0.0004	0.0003	0.0001	0.00009
**Purple (3)**	0.0009	0.0008	0.0004	0.0002	0.0001	0.00008
**Red (2)**	0.0009	0.0008	0.0001	0.00009	0.00008	0.00005
**Black (1)**	0.0009	0.0009	0.0001	0.0001	0.0001	0.0001

*calculated using the diode boundaries shown in Table S2 and S3. Data is recorded in a 50 nm nanopipette in MeCN. All simulations are performed with a rectangle function, with a smoothing factor of 300 nm. Surface Charge 2 (SC 2) refers to the surface charge in the accumulation diode at positive potential. Surface Charge (SC 3) refers to the surface charge in the depletion diode at negative potential. Surface Charge 1 (SC 1) refers to the surface charge on the rest of the nanopore walls. These are schematically represented in Figure 5A.

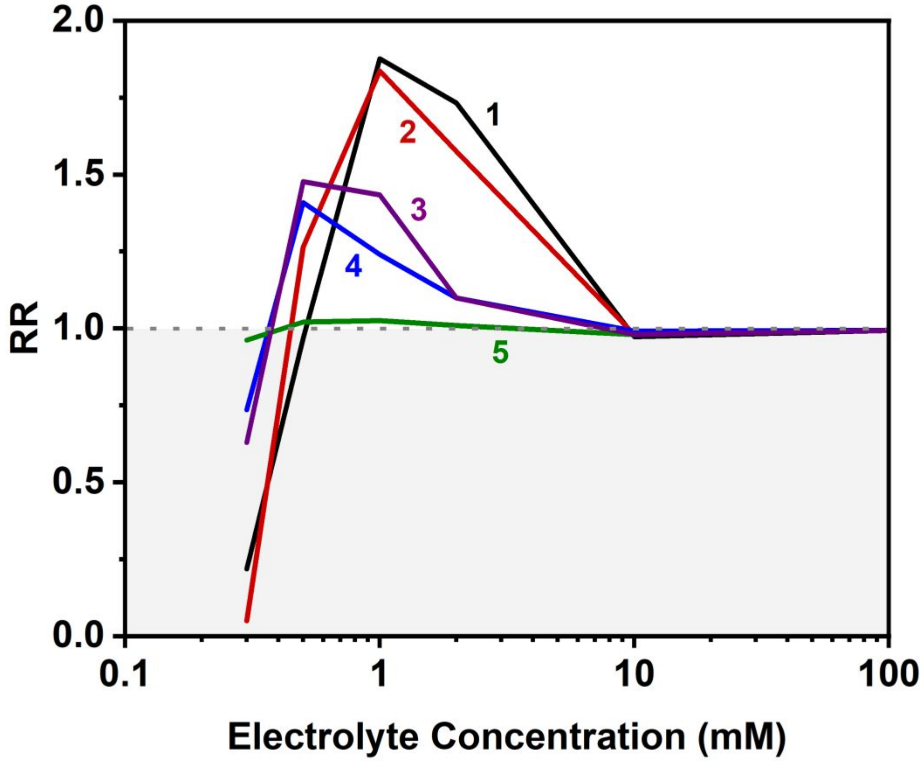

Figure S4. Theoretical calculations showing the change in rectification as a function of decreasing electrolyte concentration in MeCN, using the 5 different sets of surface charges in the accumulation region (SC2) and depletion region (SC3) shown in Table S5. The rest of the nanopore wall assumes a surface charge of 1 mC m^{-2}, and all calculations are carried out in a 50 nm pore, with a smoothing factor of 300 nm. At all surface charge values, a qualitative agreement with the experimental data is achieved, which is improved by increasing the magnitude of the surface charge in the accumulation region (SC2). Line 1 (black) is the data used in the main paper.
